# von Willebrand Factor and Oxidative Stress Parameters in Acute Coronary Syndromes

**DOI:** 10.1155/2011/918312

**Published:** 2011-08-08

**Authors:** Zoran Koprivica, Dusica Djordjevic, Milena Vuletic, Vladimir Zivkovic, Nevena Barudzic, Nebojsa Andjelkovic, Dragan Djuric, Violeta Iric-Cupic, Jelena Krkeljic, Vladimir Jakovljevic

**Affiliations:** ^1^Health Centre, 32 300 Gornji Milanovac, Serbia; ^2^Department of Physiology, Faculty of Medicine, University of Kragujevac, 34 000 Kragujevac, Serbia; ^3^Institute of Physiology “Richard Burian”, School of Medicine, University of Belgrade, 11 000 Belgrade, Serbia; ^4^University Hospital Centre “Kragujevac” and Faculty of Medicine, University of Kragujevac, 34 000 Kragujevac, Serbia

## Abstract

Considering the role of von Willebrand factor (vWf) in hemostasis, and the role of oxidative stress in the development of endothelial dysfunction and atherosclerotic disease, the aim of our study was to investigate the relationship between vWf, parameters of oxidative stress and different types of acute coronary syndromes (ACS). Levels of vWf activity (vWfAct), vWf antigen (vWfAg), nitric oxide (estimated through nitrites–NO_2_^−^), superoxide anion radical (O_2_^−^), hydrogen peroxide (H_2_O_2_), index of lipid peroxidation (estimated through thiobarbituric acid reactive substances–TBARS), superoxide dismutase (SOD) and catalase (CAT) activity of 115 patients were compared with those of 40 healthy controls. ACS patients had significantly higher vWfAct and vWfAg levels, as well as TBARS levels, while their levels of NO_2_^−^, H_2_O_2_, SOD and CAT activities were lower than controls'. vWfAg showed high specificity and sensitivity as a test to reveal healthy or diseased subjects. Multivariant logistic regression marked only vWfAg and TBARS as parameters that were under independent effect of ACS type. The results of our study support the implementation of vWf in clinical rutine and into therapeutic targets, and suggest that ACS patients are in need of antioxidant supplementation to improve their impaired antioxidant defence.

## 1. Introduction

Under physiological conditions, the vascular endothelium produces many substances that contribute importantly to hemostasis, fibrinolysis, and regulation of vessel tone and permeability [1]. One such substance is glycoprotein von Willebrand factor (vWf), which is almost exclusively produced by the endothelium, and thus is a marker of endothelial activation or dysfunction [2–4]. vWf mediates platelet adhesion to the vascular wall, platelet aggregation and serves as a plasma carrier for factor VIII, stabilizing it in the circulation [5]. Since almost all acute coronary syndromes (ACSs) result from thrombus formation in preexisting atherosclerosis [1], and given the key role of vWf in arterial thrombus formation, this biomarker attracted considerable interest as a predictor of cardiovascular disease (CVD) [1, 5]. Previously published studies suggest that there is a weak association between vWf plasma levels and risk of coronary heart disease (CHD) in general population, but its predictive value significantly rises in patients with preexisting vascular disease, diabetics, and the elderly [6–9]. It was noticed that vWf rises during the course of ACSs [4, 10, 11], and it is thought that vWf is not only a marker, but also a mediator in the pathogenesis of myocardial infarction (MI) [1]. Although a number of studies pointed out the prognostic value of vWf [12–14], there is still a long way to go before plasma vWf levels can be used as a predictor of cardiovascular disease in clinical practice [5].

von Willebrand factor can be produced and released by endothelial cells by a variety of stimuli in vitro and in vivo [5, 15, 16]. A number of substances regulate endothelial release of vWf. Among other secretion agonists and antagonists, like numerous mediators of inflammation and/or thrombosis, reactive oxygen species (ROS) play an important role. For example, superoxide anion radical (O_2_^−^) is considered to be activator of endothelial exocytosis [17], while hydrogen peroxide (H_2_O_2_) has been shown to inhibit thrombin-induced vWf secretion in a dose-dependent manner [18]. On the other hand, H_2_O_2_ induced a weak secretory response [19]. Thus, the effects of H_2_O_2_ are still to be investigated. Nitric oxide (NO) is another reactive substance that exhibits inhibitory effects on vWf secretion, but its effects are also yet to be elucidated, since the majority of research was performed on cultured endothelial cells, which may not be the ideal tool to study the effects of nitric oxide (NO) [5].

Emerging data suggest that acute presentations of coronary artery disease may involve a complex interplay between the vessel wall, inflammatory cells, and the coagulation cascade. Considering the role of vWf in hemostasis and the role of oxidative stress in the development of endothelial dysfunction and atherosclerotic disease [20–23], the aim of our study was to investigate the relationship between von Willebrand factor and parameters of oxidative stress on the one side and different types of acute coronary syndromes on the other.

## 2. Results

Demographic data of investigated groups are shown in Table 1.

The results of comparison of investigated biochemical parameters in patients and controls are shown in Figures 1, 2, 3, 4, 5, 6, 7, 8, and 9.

Results of uni- and multivariant logistic regression related to the effects of ACSs existence to changes in examined biochemical parameters are shown in Table 2. Results of univariant logistic regression marked vWfAg, vWfAct, NO_2_^−^, H_2_O_2_, TBARS, SOD, and CAT as significant. Univariant logistic regression shows the existence of differences in investigated parameters in existance of all other factors, while multivariant logistic regression marks parameters that are changed under independent effect of ACSs existance. Multivariant logistic regression did not mark any parameter as significant.

Frequency of elevated levels of vWfAct (above 163, range of referent values 48.8%–163%) and vWfAg (above 158, range of reerent values: 61.3%–158%) in ACSs patients and controls are shown in Table 3. *χ* ^2^ test showed that groups of ACSs patients and controls differed significantly in frequency of elevated levels of both vWfAct and vWfAg (*P* = 0.000 for both).

**Figure 9:**
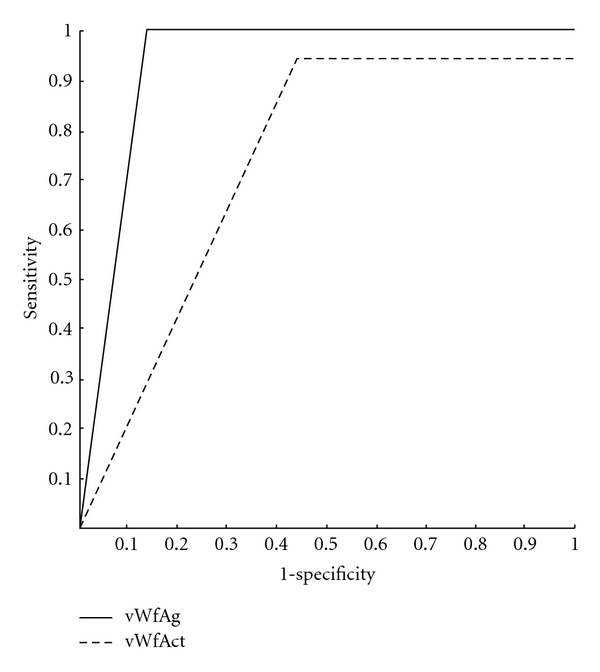
Based on the data about sensitivity and specificity of the test (measuring vWfAct and vWfAg levels), receiver operating characteristic (ROC) curve as an indicator of the discriminating ability of the vWfAct and vWfAct test was obtained. ROC curve shows that the strength of vWfAg as an indicator of ACSs existence is very high.

Based on the data presented in Table 3, sensitivity and specificity of the test (measuring vWfAct and vWfAg levels) were calculated. Sensitivity, as an indicator of the test's ability to reveal patients with disease (ACSs) was 53.91% for vWfAct and 86.0% for vWfAg, while specificity, as an indicator of test's ability to revel healthy subjects, was 97.5% for vWfAct and 100.00% for vWfAg. Receiver operating characteristic (ROC) curve (Figure 9) shows discriminative ability of the test.

Table 4 shows levels of investigated parameters in groups of patients with different type of ACSs. Results of uni- and multivariant logistic regression related to the effects of different type of ACSs existence to changes in examined biochemical parameters are shown in Table 5. Univariant logistic regression marked vWfAg, vWfAct, NO_2_^−^, H_2_O_2_, TBARS, and SOD as significant, while multivariant analysis showed that only vWfAg and TBARS were under independent effect of ACSs type. 

Table 6 shows data about investigated biochemical parameters in ACSs patients who survived and who deceased. There was no significant difference in any investigated biochemical parameter between these groups. 

Results of univariant logistic regression regarding effects of levels of biochemical parameters on outcome (survival or lethal event) of ACSs patients showed that no biochemical parameter was revealed as significant, so multivariant logistic regression was not performed (Table 7).

## 3. Discussion

Biomarkers play a pivotal role in the diagnosis and treatment of patients with cardiovascular disease [24]. Advances in our understanding of the pathophysiology of ACSs have led to the marked increase in development of biomarkers for diagnosis, risk stratification, therapeutic decision making, and assessment of clinical outcomes [25–27]. A lot of new markers measuring various components of the acute atherothrombotic event have been described, and novel biomarkers of endothelial activation, inflammation, coagulation, and platelet activation are intensively investigated [28–31]. Among other perspective prognostic biomarkers that have not been incorporated into routine clinical use yet, von Willebrand factor rises considerable attention. 

Many different vWf-dependent laboratory assays have been developed to correctly diagnose and classify von Willebrand disease [32]. However, when investigating vWf as a risk factor for cardiovascular disease, assays for plasma vWf antigen and plasma vWf activity are used in most cases [1]. ACSs patients in our study had significantly elevated levels of both vWfAct and vWfAg compared with healthy control subjects. Levels of vWfAct did not differ significantly among patients with different type of ACSs; vWfAct was elevated only in STEMI (ST elevation MI) and NSTEMI (non-ST elevation MI) ACSs patients, while patients with unstable angina had vWfAct levels similar to control subjects. Regarding vWfAg, there were significant differences in its level among patients with different types of ACSs as well as between controls and STEMI and NSTEMI patients. Only vWfAg levels of patients with UA did not differ from controls. The results of our study are in consent with previously reported data. Spiel et al. [1] and Paulinska et al. [16] reported that the published vWf data [10, 11, 33–37] shows that patients with acute myocardial infarction (AMI) have markedly increased vWf values compared with unstable angina (UA) and coronary artery disease (CAD) patients as well as compared with healthy control subjects. That vWf is biochemical parameter that distinguishes well between the healthy and people with ACSs our study proved not only by assessing differences in its levels between ACSs patients and controls, but also by comparing the frequency of the existance of elevated vWf levels in these two groups as well as by counting its specificity and sensitivity. Sensitivity of vWfAg, as an indicator of its ability to reveal patients with disease, was 86%, while its specificity, as an indicator of its ability to reveal healthy subjects, was 100%. Sensitivity of vWfAct was significantly lower, but its specificity was also very high (97.5%). As it can also be seen from Figure 9 that shows ROC curves for vWfAct and vWfAg, the strength of vWfAg as an indicator of ACSs existance is very high. Results of logistic regression also highlighted this parameter as significant—univariant logistic regression showed that type of ACSs affects both vWfAct and vWfAg levels, but multivariant regression marked only vWfAg as the one whose change is under independent effect of ACSs type not due to existance of other cofactors.

The results of previous studies regarding prognostic value of vWf for cardiovascular events are equivocal. Many studies found increased relative risk in subjects with the highest levels of vWf [6, 38, 39], but in the majority of studies, the association between vWf and CHD risk disappeared after adjustment for conventional risk factors [8, 9]. However, in contrast to results of studies performed in the general population, in patients with preexisting vascular disease, vWf revealed as significantly predictive parameter for adverse cardiac events, including death [13, 14, 40–42]. Our study was not prospective, but since 4 patients died few days after admission, we compared their admission levels of investigated biochemical parameters with levels of patients that survived. No statistical difference was found, neither in vWfAct and vWfAg levels, nor in levels of redox parameters.

The second part of our investigation related to the assessment of subjects' redox state. Oxidative stress has been suggested to play an important role in the development of more than two hundred acute and chronic human diseases as well as in aging [21–23, 43–46]. The results of our study showed that ACSs patients had significantly less efficient antioxidative defence system compared with controls (significantly lower levels of SOD and CAT activity) which resulted in significantly increased levels of lipid peroxidation. Furthermore, ACSs patients had significantly lower levels of nitric oxide (nitrites), which is an indicator of endothelial dysfunction. Suprisingly, H_2_O_2_ levels of ACSs patients were significantly lower than controls'. The fact that ACSs patients had lower NO and H_2_O_2_ levels may be brought into connection with vWf levels, since reactive oxygen species and NO are involved in the regulation of vWf release. It was previously suggested that the blockade of nitric oxide enhances the stimulated release of vWf in humans [47, 48], that is, NO exerts inhibitory effects on vWf release [5]. So, is thought for H_2_O_2_ [5]. ACSs patients were deficient in NO and H_2_O_2_ compared with controls, so NO and H_2_O_2_ inhibition of vWf release was probably lower. When comparing oxidative status of patients with different ACSs types, only SOD differed between STEMI and UA patients (UA patients had lower SOD activity). On the other hand, whenever the difference among 4 groups (3 ACSs groups and control group; Table 4) was found, it related to differences between every group of ACSs patients and controls. These results suggest that ACSs patients are under higher oxidative stress compared with control subjects, no matter which type of ACSs they have. However, although ACSs patients differed from controls in a few oxidative stress parameters, only TBARS was found to be under the independent effect of ACSs type.

The results of our study point out to von Willebrand factor antigen as strong indicator of existance of any kind of ACSs. This supports the implementation of vWf in clinical routine and into therapeutic targets. Results related to the investigation of oxidative stress in ACSs patients suggest that antioxidant supplementation is needed to improve the antioxidant defence, since enzymatic antioxidant defense is compromised and lipid peroxidation consequently increased in those patients.

## 4. Patients and Methods

### 4.1. Subjects

The research was carried out within a group of 115 patients who were consecutively admitted to the Intensive Care Unit of University Hospital Center “Kragujevac”, Serbia. All patients met the criteria for ACSs diagnosis (the presence of two out of these three criteria is enough to diagnose ACSs): (1) chest pain, (2) electrocardiographic changes (ST elevation or depression ≥1 mm, or T wave inversion), and (3) serum cardiac markers changes (creatine kinase (CK), creatine kinase MB (CK-MB), and troponin T (TnT)) [49]. The diagnosis of STEMI was defined as the concurrence of prolonged chest pain or discomfort with persistent ST-segment elevation of greater than 1 mm in 2 or more contiguous leads or with presumed new left bundle-branch block with cardiac enzymes (total creatine kinase and creatine kinase MB fraction) above twice the upper normal limit [50]. The diagnosis of NSTEMI included the presence of typical angina at rest associated with acute and transient ST-segment or T-wave changes with cardiac enzymes above twice the upper normal limit, raised troponin I levels to at least “high risk” values (>0.6 ng/mL), or both [50]. Patients with clinical or electrocardiographic (ECG) features of non-STEMI but with normal cardiac enzymes plus normal troponin levels were classified as UA [50].

The study was approved by Ethical committee of University Hospital Center “Kragujevac”, Serbia.

### 4.2. Protocol

After admission to hospital patients were taken a blood sample from which von Willebrand factor activity (vWfAct), von Willebrand antigen (vWfAg), levels of nitric oxide (NO), superoxide anion radical (O_2_^−^), hydrogen peroxide (H_2_O_2_), index of lipid peroxidation (TBARS), superoxide dismutase activity (SOD), and catalase activity (CAT) were determined.

### 4.3. Biochemical Assays

Blood samples were taken from the antecubital veins into Vacutainer test tube containing sodium citrate anticoagulant. Blood was centrifuged to separate plasma and red blood cells (RBCs).

### 4.4. von Willebrand Factor Activity and von Willebrand Factor Antigen Determination

Determination of vWfAct and vWfAg was performed using commercial assay kit (HemosIL 0020004700 and 0020002300) on ACL Elite Pro apparatus manufactured by Instrumentation Laboratory, Bedford, Mass, USA. vWfAct and vWfAg results are reported in % of normality.

### 4.5. Nitric Oxide Determination

Nitric oxide (NO) decomposes rapidly to form stable metabolite nitrite/nitrate products. Nitrite (NO_2_^−^) was determined as an index of nitric oxide production with Griess reagent [51]. 0.1 mL 3N PCA (Perchloride acid), 0.4 mL 20 mM EDTA (ethylenediaminetetraacetic acid), and 0.2 mL plasma were put on ice for 15 min then centrifuged 15 min at 6000 rpm. After pouring off the supernatant, 220 *μ*L K_2_CO_3_ was added. Nitrites were measured at 550 nm. Distilled water was used as a blank probe.

### 4.6. Superoxide Anion Radical Determination

 The level of superoxide anion radical (O_2_^−^) was measured using NBT (nitro blue tetrazolium) reaction in TRIS-buffer combined with plasma samples and read at 530 nm [52]. 

### 4.7. Hydrogen Peroxide Determination

The protocol for measurement of hydrogen peroxide (H_2_O_2_) is based on oxidation of phenol red in the presence of horseradish peroxidase [53]. Two hundred *μ*L sample with 800 *μ*L PRS (phenol red solution) and 10 *μ*L POD (horse radish peroxidase) were combined (1 : 20). The level of H_2_O_2_ was measured at 610 nm.

### 4.8. Index of Lipid Peroxidation (Thiobarbituric Acid Reactive Substances, TBARS)

The degree of lipid peroxidation in plasma was estimated by measuring of thiobarbituric acid reactive substances (TBARS) using 1% TBA (Thiobarbituric Acid) in 0.05 NaOH, incubated with plasma at 100°C for 15 min and read at 530 nm. Distilled water was used as a blank probe. TBA extract was obtained by combining 0.8 mL plasma and 0.4 mL TCA (trichloro acetic acid), then samples were put on ice for 10 minutes and centrifuged for 15 min at 6000 rpm. This method was described previously [54]. 

### 4.9. Determination of Antioxidant Enzymes

Isolated RBCs were washed three times with 3 volumes of ice-cold 0.9 mmol/L NaCl, and hemolysates containing about 50 g Hb/L (prepared according to McCord and Fridovich [55]) were used for the determination of catalase (CAT) activity. CAT activity was determined according to Beutler [56]. Lysates were diluted with distilled water (1 : 7 v/v) and treated with chloroform-ethanol (0.6 : 1 v/v) to remove haemoglobin [57]. Then, 50 *μ*L catalase buffer, 100 *μ*L sample, and 1 mL 10 mM H_2_O_2_ were added to the samples. Detection was performed at 360 nm. Distilled water was used as a blank probe. Superoxide dismutase (SOD) activity was determined by the epinephrine method of Misra and Fridovich [58]. A hundred *μ*L lysate and 1 mL carbonate buffer were mixed, and then, 100 *μ*L of epinephrine was added. Detection was performed at 470 nm.

### 4.10. Statistics

The statistical analysis was performed with SPSS 10.0 for Windows. Results are expressed as the means ± standard deviation (median). Data on figures is presented as mean + standard deviation. After checking data distribution, the appropriate parametric or nonparametric test was used. The differences between two groups were assessed using *t*-test or Mann-Whitney test, while the differences between more than three groups were assessed using one-way ANOVA or Kruskal-Wallis test. For ANOVA posthoc analysis, Bonferroni test was used. To define the parameters that mostly change in ACSs, univariant logistic regression was used. Parameters that were marked as significant in univariant logistic regression entered multivariant logistic regression. Multivariant logistic regression marked parameters that were under independent effect of ACSs. *χ* ^2^ test was used to assess the difference in frequency of elevated levels of vWfAct and vWfAg. Based on the data about this frequency, sensitivity and specificity of the test (vWfAct and vWfAg test) were calculated. Based on the data about sensitivity and specificity of the test, the receiver operating characteristics (ROC) curve was determined.

## Figures and Tables

**Figure 1:**
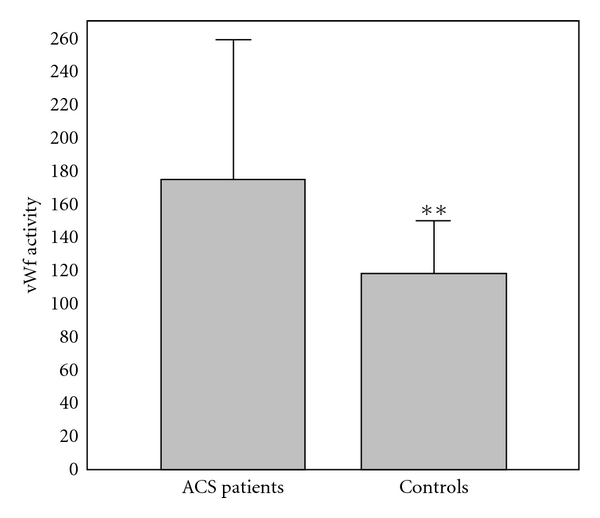
Values of von Willebrand factor activity (%) in group of ACSs patient and in group of control subjects (mean + SD). Compared with control subjects, ACSs patients had statistically higher levels of vWfAct (175.27 ± 86.52 (166.0) % compared with 118.35 ± 33.11 (122.0) %; Mann-Whitney *U* test; **P < 0.01).

**Figure 2:**
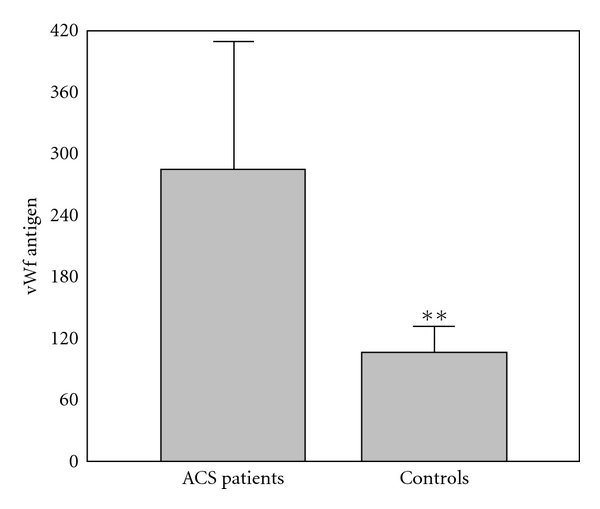
Values of von Willebrand factor antigen (%) in group of ACSs patient and in group of control subjects (mean + SD). Compared with control subjects, ACSs patients had statistically higher levels of vWfAg (284.82 ± 126.66 (261.0) % compared with 104.57 ± 25.52 (108.0) %; *t*-test; **P < 0.01).

**Figure 3:**
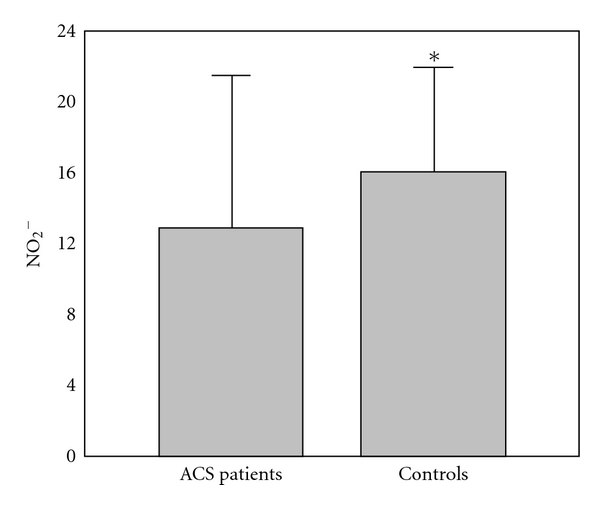
Values of nitric oxide (nitrites) levels (nmol/mL) in group of ACSs patient and in group of control subjects (mean + SD). Compared with control subjects, ACSs patients had statistically lower levels of NO_2_^−^ (12.88 ± 8.40 (10.52) nmol/mL compared with 15.98 ± 6.05 (17.20) nmol/mL; Mann-Whitney *U* test; *P < 0.05).

**Figure 4:**
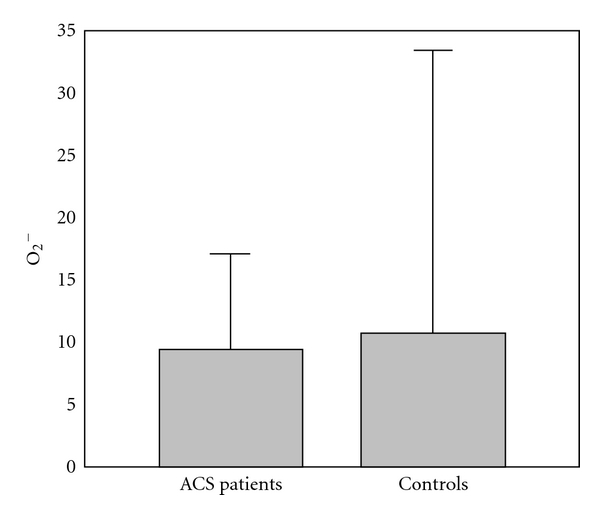
Values of superoxide anion radical levels (nmol/mL) in group of ACSs patient and in group of control subjects (mean + SD). ACSs patients and controls did not differ in levels of O_2_^−^ (9.34 + 7.83 (7.58) nmol/mL compared with 10.63 + 22.90 (5.27); Mann Whitney *U* test; *P* > 0.05).

**Figure 5:**
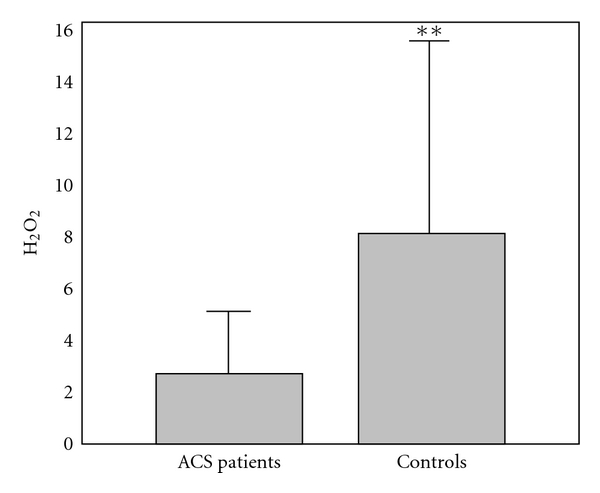
Values of hydrogen peroxide (nmol/mL) in group of ACSs patient and in group of control subjects (mean + SD). Compared with control subjects, ACSs patients had statistically lower levels of H_2_O_2_ (2.75 ± 2.56 (2.12) nmol/mL compared with 8.11 ± 7.52 (5.21); Mann Whitney *U* test; **P < 0.01).

**Figure 6:**
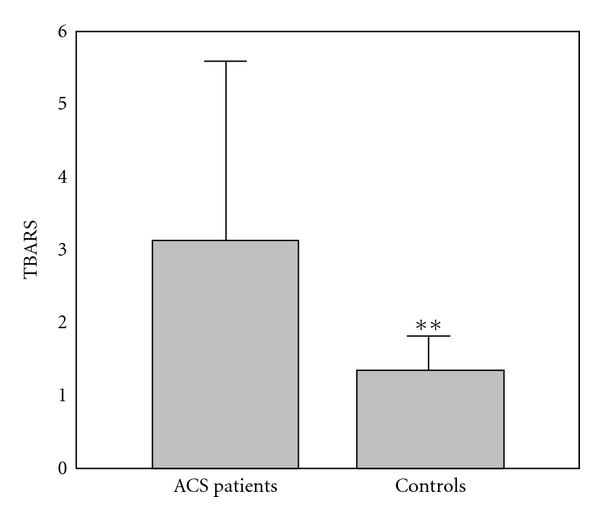
Values of index of lipid peroxidation (TBARS) levels (*μ*mol/mL) in group of ACSs patient and in group of control subjects (mean + SD). Compared with control subjects, ACSs patients had statistically higher TBARS compared with control subjects (3.12 ± 2.45 (2.48) U/g Hb × 10^3^ compared with 1.34 ± 0.47 (1.19) U/g Hb × 10^3^; *t*-test; **P < 0.01).

**Figure 7:**
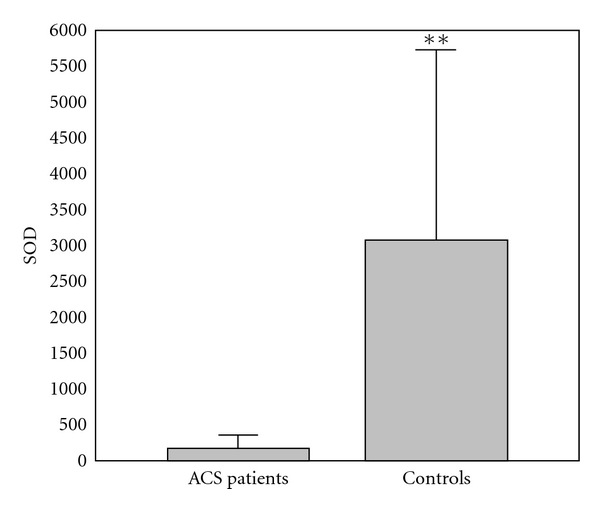
Values of superoxide dismutase activity (U/g Hb × 10^3^) in group of ACSs patient and in group of control subjects (mean + SD). Compared with control subjects, ACSs patients had statistically lower SOD activity (156.83 ± 140.94 (89.54) U/g Hb × 10^3^ compared with 3078.21 ± 2664.35 (2433.71) U/g Hb × 10^3^; Mann-Whitney *U* test; **P < 0.01).

**Figure 8:**
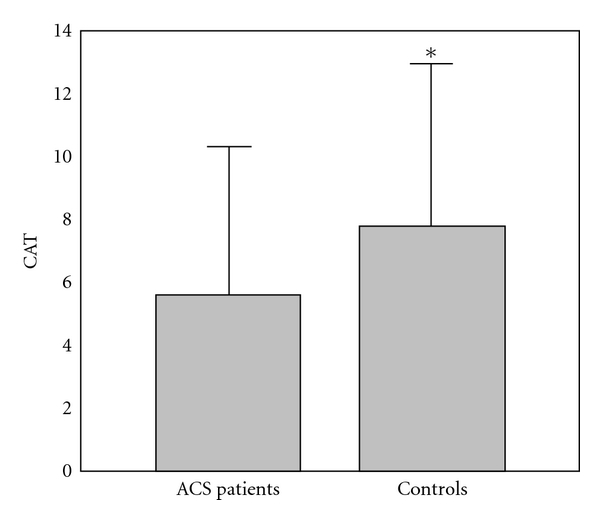
Values of catalase activity (U/g Hb × 10^3^) in group of ACSs patient and in group of control subjects (mean + SD). Compared with control subjects, ACSs patients had statistically lower CAT activity (5.55 ± 4.99 (4.00) U/g Hb × 10^3^ compared with 7.80 ± 5.10 (6.87) U/g Hb × 10^3^; Mann-Whitney *U* test; *P < 0.05).

**Table 1:** Demographic characteristics of investigated groups.

Parameters	ACSs patients	Controls
Number of patients (*n*)	115	40
Sex (male/female)	74/41	26/14
Age (*X* ± SD years)	67.0 ± 8.0	65.0 ± 3.0

**Table 2:** Results of uni- and multivariant logistic regression related to the effects of ACSs existence to changes in examined biochemical parameters (exp*B*—relative risk; CI—confidence interval; *P < 0.005; **P < 0.001).

Parameter	Univariant logistic regression		Multivariant logistic regression	
	exp*B* (95% CI)	Sig.	exp*B* (95% CI)	Sig.
vWfAct	0.988 (0.981–0.994)	*P* = 0.000**	1.033 (0.984–1.084)	*P* = 0.193
vWfAg	0.962 (0.947–0.976)	*P* = 0.000**	0.912 (0.810–1.027)	*P* = 0.130
NO_2_^−^	1.062 (1.012–1,114)	*P* = 0.014**	1.010 (1.002–1.114)	*P* = 0.914
O_2_^−^	1.005 (0.983–1.028)	*P* = 0.643	—	—
H_2_O_2_	1.254 (1.124–1.400)	*P* = 0.000**	1.054 (1,024–1.300)	*P* = 0.988
TBARS	0.399 (0.251–0.635)	*P* = 0.000**	0.999 (0.990–1.100)	*P* = 0.999
SOD	1.007 (1.004–1.011)	*P* = 0.000**	1.017 (1.004–1.031)	*P* = 0.899
CAT	1.046 (0.980–1.117)	*P* = 0.174	—	—

**Table 3:** Frequencies of elevated levels of vWfAct and vWfAg in ACSs patients and controls.

		Group		Sig.
		ACSs	Controls	
vWfAg	Elevated levels	99 (86.1%)	0 (0.0%)	*P* = 0.000**
	Normal levels	16 (13.9%)	40 (100.0%)	
vWfAct	Elevated levels	62 (53.9%)	1 (2.5%)	*P* = 0.000**
	Normal levels	53 (46.1%)	39 (97.5%)	

**Table 4:** Levels of investigated biochemical parameters in different subgroups of subjects.

	*X* ± SD (median)	Sig.
vWfAct (%)		

STEMI (*n* = 65)	185.36 ± 95.25 (170.00)	Kruskal-Wallis test: *P* = 0.000** *Mann-Whitney U test: *
NSTEMI (*n* = 36)	167.92 ± 75.56 (168.50)	STEMI versus NSTEMI *P* = 0.173 STEMI versus UA *P* = 0.072
UA (*n* = 14)	130.48 ± 50.34 (121.00)	STEMI versus Control *P* = 0.000** NSTEMI versus UA *P* = 0.214
Control (*n* = 40)	118.35 ± 33.11 (122.00)	NSTEMI versus Control *P* = 0.001** UA versus Control *P* = 0.819

vWfAg (%)		

STEMI	317.21 ± 139.50 (302.00)	ONE way ANOVA: *P* = 0.000** *Bonferroni: *
NSTEMI	256.40 ± 95.23 (229.25)	STEMI versus NSTEMI *P* = 0.012* STEMI versus UA *P* = 0.000**
UA	169.27 ± 52.47 (181.50)	STEMI versus Control *P* = 0.000** NSTEMI versus UA *P* = 0.021*
Control	104.57 ± 25.52 (108.00)	NSTEMI versus Control *P* = 0.000** UA versus Control *P* = 0.991

O_2_^−^ (nmol/mL)		

STEMI	8.90 ± 6.91 (7.25)	Kruskal-Wallis test: *P* = 0.284
NSTEMI	9.68 ± 9.45 (7.58)	
UA	10.38 ± 0.57 (10.54)	
Control	10.63 ± 22.90 (5.27)	

H_2_O_2_ (nmol/mL)		

STEMI	2.70 ± 1.66 (2.14)	Kruskal-Wallis test: *P* = 0.000** *Mann-Whitney U test: *
NSTEMI	2.41 ± 2.04 (1.98)	STEMI versus NSTEMI *P* = 0.181 STEMI versus UA *P* = 0.235
UA	5.06 ± 7.37 (1.66)	STEMI versus Control *P* = 0.000** NSTEMI versus UA *P* = 0.872
Control	8.11 ± 7.52 (5.21)	NSTEMI versus Control *P* = 0.000** UA versus Control *P* = 0.000**

NO_2_^−^ (nmol/mL)		

STEMI	13.16 ± 7.57 (12.65)	ONE way ANOVA: *P* = 0.081
NSTEMI	13.12 ± 9.62 (9.69)	
UA	9.54 ± 6.95 (7.22)	
Control	15.98 ± 6.05 (17.20)	

TBARS (*μ*mol/mL)		

STEMI	3.42 ± 2.61 (2.69)	Kruskal-Wallis test: *P* = 0.000** *Mann-Whitney U test: *
NSTEMI	2.96 ± 2.47 (2.21)	STEMI versus NSTEMI *P* = 0.999 STEMI versus UA *P* = 0.999
UA	2.10 ± 0.56 (2.30)	STEMI versus Control *P* = 0.000** NSTEMI versus UA *P* = 0.999
Control	1.34 ± 0.47 (1.19)	NSTEMI versus Control *P* = 0.000** UA versus Control *P* = 0.000**

SOD (U/g Hb ×10^3^)		

STEMI	185.04 ± 154.82 (124.54)	Kruskal-Wallis test: *P* = 0.000** *Mann-Whitney U test: *
NSTEMI	140.03 ± 129.84 (87.91)	STEMI versus NSTEMI *P* = 0.069 STEMI versus UA *P* = 0.032*
UA	67.20 ± 20.95 (66.34)	STEMI versus Control *P* = 0.000** NSTEMI versus UA *P* = 0.615
Control	3078.21 ± 2664.35 (2433.71)	NSTEMI versus Control *P* = 0.000** UA versus Control *P* = 0.000**

CAT (U/g Hb ×10^3^)		

STEMI	5.94 ± 5.94 (3.25)	Kruskal-Wallis test: *P* = 0.159
NSTEMI	5.30 ± 4.14 (4.37)	
UA	4.38 ± 2.65 (4.12)	
Control	7.80 ± 5.10 (6.87)	

**Table 5:** Results of uni- and multivariant logistic regression related to the effects of different type ACSs to changes in examined biochemical parameters (B—unstandardized regression coefficient; CI—confidence interval; *P < 0.005; **P < 0.001).

Parameter	Univariate logistic regression		Multivariate logistic regression	
	*B* (95% CI)	Sig.	*B* (95% CI)	Sig.
vWfAct	−0.006 (−0.008–(−0.003))	*P* = 0.000**	0.000 (−0.004–0.003)	*P* = 0.948
vWfAg	−0.006 (−0.007–(−0.005))	*P* = 0.000**	−0.004 (−0.006–(−0.001))	*P* = 0.007**
NO	0.027 (0.001–0.053)	*P* = 0.038*	−0.002 (−0.026–0.023)	*P* = 0.897
O_2_	0.003 (−0.010–0.016)	*P* = 0.648	—	—
H_2_O_2_	0.099 (0.064–0.133)	*P* = 0.000**	0.072 (0.013–0.131)	*P* = 0.018
TBARS	−0.161 (−0.220–(−0.102))	*P* = 0.000**	−0.100 (−0.183–(−0.017))	*P* = 0.019*
SOD	0.000 (0.000–0.000)	*P* = 0.000**	0.001 (0.000–0.001)	*P* = 0.262
CAT	0.015 (−0.024–0.053)	*P* = 0.450	—	—

**Table 6:** Levels of investigated biochemical parameters in ACSs patients who survived and who deceased.

Subjects	*X* ± SD (median)	Sig.
vWfAct (%)		

Survivors (*n* = 111)	172.00 ± 83.94 (168.00)	Mann-Whitney *U* test, *P* = 0.708
Deceased (*n* = 4)	189.80 ± 105.94 (175.00)	

vWfAg (%)		

Survivors	274.86 ± 120.70 (241.00)	Mann-Whitney *U* test, *P* = 0.119
Deceased	395.30 ± 182.16 (416.00)	

NO_2_^−^ (nmol/mL)		

Survivors	12.68 ± 7.86 (12.32)	*t*-test, *P* = 0.998
Deceased	12.67 ± 10.28 (8.18)	

O_2_^−^ (nmol/mL)		

Survivors	9.15 ± 10.68 (6.59)	Mann-Whitney *U* test, *P* = 0.150
Deceased	15.42 ± 28.24 (3.62)	

H_2_O_2_ (nmol/mL)		

Survivors	2.99 ± 3.53 (2.20)	Mann-Whitney *U* test, *P* = 0.918
Deceased	2.16 ± 0.56 (2.05)	

TBARS (*μ*mol/mL)		

Survivors	4.26 ± 3.39 (2.95)	Mann-Whitney *U* test, *P* = 0.394
Deceased	5.24 ± 3.24 (5.06)	

SOD (U/g Hb × 10^3^)		

Survivors	157.55 ± 125.22 (107.45)	Mann-Whitney *U* test, *P* = 0.836
Deceased	175.82 ± 198,86 (118.03)	

CAT (U/g Hb × 10^3^)		

Survivors	6.16 ± 5.22 (4.00)	Mann-Whitney *U* test, *P* = 0.073
Deceased	11.30 ± 7.36 (10.00)	

**Table 7:** Univariant logistic regression regarding effects of biochemical parameters on outcome of ACSs patients (exp*B*—relative risk; CI—confidence interval).

Parameter	Univariant logistic regression	
	exp*B* (95% CI)	Sig.
vWfAct	1.002 (0.992–1.013)	*P* = 0.647
vWfAg	1.006 (1.000–1.013)	*P* = 0.052
NO_2_^−^	1.000 (0.892–1.121)	*P* = 0.998
O_2_	1.026 (0.979–1.075)	*P* = 0.291
H_2_O_2_	0.846 (0.447–1.599)	*P* = 0.606
TBARS	1.083 (0.843–1.391)	*P* = 0.531
SOD	1.001 (1.995–1.008)	*P* = 0.757
CAT	1.120 (0.995–1.262)	*P* = 0.061

## Abbreviations

ACSs: Acute coronary syndromes
STEMI: ST-segment elevation myocardial infarction
NSTEMI: Non-ST-segment elevation myocardial infarction
UA: Unstable angina
vWfAct: von Willebrand factor activity
vWfAg: von Willebrand factor antigen
SOD: Superoxide dismutase
CAT: Catalase
ROS: Reactive oxygen species
CVD: Cardiovascular disease
CHD: Coronary heart disease
ROC: Receiver operating characteristics.
